# Longitudinal comparison of HIV-1 plasma viral load and cellular proviral load

**DOI:** 10.7448/IAS.17.4.19669

**Published:** 2014-11-02

**Authors:** Finja Schweitzer, Visa Mikkola, Monika Timmen-Wego, Björn Jensen, Gerd Fätkenheuer, Rolf Kaiser

**Affiliations:** 1 Institute of Virology, University of Köln, Köln, Germany; 2 Department of Gastroenterology and Hepatology, University Hospital Düsseldorf, Düsseldorf, Germany; 3 Department of Internal Medicine, University Hospital Köln, Köln, Germany

## Abstract

**Introduction:**

The goal of antiretroviral treatment (ART) in HIV-1 infection is the permanent suppression of plasma viral load (pVL) below the currently existing limit of detection of 50 copies/mL (DAIG HIV-therapy guidelines). Therefore, treatment effectiveness is based on pVL. pVL measurements however do not give any information about the viral reservoir in peripheral blood mononuclear cells (PBMCs). Therefore, the proviral DNA of HIV-1 could be a useful marker for the investigation of viral reservoirs and monitoring ART in patients showing undetectable pVL.

**Materials and Methods:**

Seventy-seven treatment-experienced HIV-1 infected patients with pVL <50 copies/mL were randomly selected. pVL and proviral DNA load were measured using the Roche COBAS TaqMan HIV-1 v2.0 assay. Additionally, CD4+ cell count per mL and the total white blood cell (wbc) count per mL were determined for each patient. Follow-up data were collected 24 weeks after the time point of the first measurement. Proviral DNA load per mL, CD4+ cell count per mL as well as wbc count per mL were observed over time and differences were estimated using the Mann–Whitney U test. Additionally, correlations between the clinical parameters were analyzed using the two-sided Pearson correlation analysis.

**Results:**

Out of the 77 patients, 38 show a significant increase in proviral load per mL over time (p=0,001), whereas 39 patients show a significant decrease (p=0,001). No differences became visible in the CD4+ cell count per mL comparing week 0 and week 24 data. Thirty five patients show a significant increase in wbc count per mL over time (p<0,001), while 42 patients show a significant decrease (p<0,001). A significant correlation of increasing proviral load per mL and wbc count per mL for data of the first (p<0.001) and the second measurement (p<0.001) can be detected, while there are no correlations found between proviral load per mL and CD4+ cell count per mL.

**Conclusions:**

Our data show that the presence of viral reservoirs in other cell types and not only CD4+ cells is most probable. HIV-1 proviral DNA seems to be an interesting marker in patients with undetectable pVL and allows the assessment of replication under ART. Nevertheless, further longitudinal studies are needed to assess the usefulness and the clinical significance of this marker.

